# Inflammation and B cell activation define a plasma proteome signature predicting tuberculosis in people with HIV

**DOI:** 10.1128/mbio.01585-25

**Published:** 2025-08-28

**Authors:** Katharina Kusejko, Mohammad Arefian, Diane Duroux, Marius Zeeb, Cédric Dollé, Matthias Hoffmann, Niklaus Labhardt, Gilles Wandeler, Matthias Cavassini, Sabine Haller, Enos Bernasconi, Doris Russenberger, Roger D. Kouyos, Huldrych F. Günthard, Ben C. Collins, Johannes Nemeth, the Swiss HIV Cohort Study

**Affiliations:** 1 Department of Infectious Diseases and Hospital Epidemiology, University Hospital Zurich and University of Zurich 261116 , Zürich, Switzerland; 2 Institute of Medical Virology, University of Zurich 27217, Zürich, Switzerland; 3 School of Biological Sciences, Queen's University Belfast 98871, Belfast, United Kingdom; 4 ETH Zurich, AI Center 30806 , Zürich, Switzerland; 5 Department of Biosystems Science and Engineering, ETH Zurich 211122 , Basel, Switzerland; 6 Department of Quantitative Biomedicine, University of Zurich 726932, Zürich, Switzerland; 7 Division of Infectious Diseases and Hospital Epidemiology, Cantonal Hospital Olten, Olten, Switzerland; 8 Division of Clinical Epidemiology, Department of Clinical Research, University Hospital Basel 718239 , Basel, Switzerland; 9 University of Basel 27209, Basel, Switzerland; 10 Division of Infectious Diseases, Bern University Hospital, University of Bern 27210, Bern, Switzerland; 11 Division of Infectious Diseases, University Hospital of Lausanne 27217, Lausanne, Switzerland; 12 Division of Infectious Diseases, Infection Prevention and Travel Medicine, HOCH Health Ostschweiz 718239 , St. Gallen, Switzerland; 13 Division of Infectious Diseases, Ente Ospedaliero Cantonale 27219, Lugano, Switzerland; 14 University of Geneva and University of Southern Switzerland, Lugano, Switzerland; Dana-Farber Cancer Institute, Boston, Massachusetts, USA

**Keywords:** HIV/TB co-infection, predicting tuberculosis, machine learning, proteomics

## Abstract

Improved biomarkers for predicting progression to active tuberculosis (TB) are urgently needed, especially in people with HIV, who are at elevated risk. We used high-throughput plasma proteomics and machine learning to identify signatures associated with TB progression in this population. From the Swiss HIV Cohort Study, we analyzed plasma samples collected at least 6 months before TB diagnosis from 91 participants who later developed TB. We selected 293 controls matched for demographic and clinical parameters who remained TB-free to achieve a risk score specific to active TB. In total, 583 samples were analyzed, with 613–1,283 proteins quantified per sample. A random forest classifier predicted a significantly higher median probability of TB progression for cases (33%) than for controls (16%; *P* < 0.001). In this matched population, the score achieved an area under the receiver-operating characteristic curve of 0.77, an area under the precision-recall curve (AUPRC) of 0.60 (as compared to an expected AUPRC of 0.29), as well as a specificity of 87.3% and a sensitivity of 58.6% using the optimal threshold of 0.311. The plasma proteome of individuals who progressed to active TB showed a distinct shift toward systemic inflammation, B cell activation, and immunoglobulin production. Independent of progression to active TB, the proteome score correlated with broader indicators of immune suppression, including lower CD4 counts and unsuppressed HIV RNA. This suggests that integrating proteomic and clinical data could enhance the overall predictive power of the score.

**IMPORTANCE:**

We still lack reliable tools to predict who will develop tuberculosis (TB) among people with HIV. Moreover, the underlying biological events driving progression remain poorly understood. Our study reveals early immune changes that include unexpected alterations in B cell activation and antibody responses. These findings suggest that humoral immunity may play a more important role in TB pathogenesis than previously recognized and offer promising new directions for biomarker discovery and targeted prevention.

## INTRODUCTION

Active tuberculosis (TB) remains a leading cause of death from an infectious agent, with around 1.25 million deaths globally in 2023 (1). While it is estimated that around 28% of the global population has been exposed to *Mycobacterium tuberculosis* (MTB), fewer than 10% of infected individuals progress to active TB (2). Predicting progression to active TB is thus critical toward identifying individuals at highest risk and allocating preventive measures effectively. A key milestone in achieving this goal, also integrated into the World Health Organization’s strategy to end the global TB epidemic, involves developing reliable biomarkers to diagnose and predict active TB (3).

People with human immunodeficiency virus (HIV) (PWH) are disproportionately affected by the TB epidemic (4). HIV accelerates the progression to active TB, and predicting active TB in PWH is particularly challenging. Current immunological tests for MTB infection, such as the tuberculin skin test (TST) and interferon-gamma release assays (IGRA), depend on a functional immune system and specifically rely on CD4+ T cells. Since HIV-1 targets CD4+ T cells, leading to their depletion and dysfunction, these tests often perform poorly in PWH. Even with antiretroviral therapy (ART) and CD4+ T cell recovery, the MTB-specific CD4+ T cell repertoire remains compromised in most individuals (5, 6). Since the risk for the emergence of active TB is highest within the first 2 years following MTB exposure, developing predictive tools that identify progression toward active TB within the critical first 2-to-4-year window would enable more targeted preventive strategies (7, 8).

**Fig 1:**
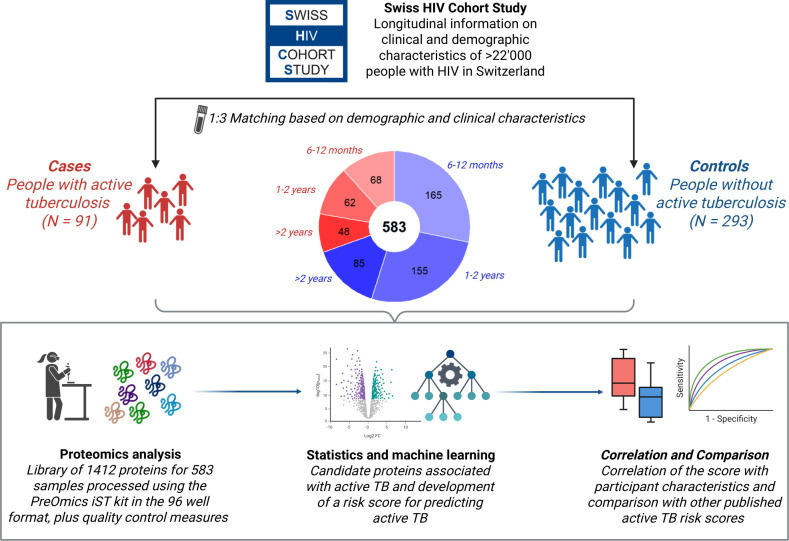
Representation of the workflow toward generating a protein risk score for developing active tuberculosis. Created in BioRender. Kusejko, K. (2025) (https://BioRender.com/ske23uw).

This project utilizes high-throughput quantitative proteomics analysis of samples collected in the Swiss HIV Cohort Study (SHCS). Samples from PWH who developed active TB were compared with matched controls (i.e., PWH who did not develop active TB). Using machine learning techniques, we developed a risk score for active TB progression and analyzed its association with other participant characteristics. The workflow for generating and validating the protein-based risk score is visualized in Fig. 1.

## MATERIALS AND METHODS

### Swiss HIV Cohort Study (SHCS)

The SHCS launched in 1988 is a multicentric cohort study enrolling adult PWH in Switzerland (9). In biannual follow-up visits, in-depth clinical, demographic, and laboratory information are collected. In addition, plasma and peripheral blood mononuclear cell (PBMC) samples are collected routinely for each participant on a yearly basis. According to standard care guidelines, a tuberculosis test (either TST or IGRA) is performed once at the SHCS enrollment for every participant. The SHCS was approved by the ethics committees of the participating institutions (BASEC-Nr. 2023-02080), and all participants provided written informed consent.

### Definitions

Active tuberculosis is recorded as follows: (i) *definitive pulmonary tuberculosis*: confirmed by direct detection (i.e., PCR approaches or culture of pulmonary sample); (ii) *presumptive pulmonary tuberculosis*: by detection of acid-fast bacilli in sputum without confirmation by culture or PCR and response to specific treatment, or suggestive infiltrate on chest X-ray and anamnestic exposure to TB and response to specific treatment; (iii) *definitive extrapulmonary tuberculosis*: by culture of non-pulmonary sample; and (iv) *presumptive extrapulmonary tuberculosis*: by the detection of acid-fast bacilli in stool, blood, bodily fluids, or tissue or through histological analysis of lymph nodes, where the mycobacterial species cannot be identified by culture. This diagnosis is further supported by the presence of a concurrent confirmed diagnosis of pulmonary tuberculosis or by a clinical response to standard anti-tuberculosis treatment (8–10).

### Selection of the study population

All SHCS participants with a diagnosis of active TB and available plasma samples between 6 months and 4 years before diagnosis were included in this study. A control population (i.e., SHCS participants without diagnosis of active TB and without reported preventive treatment against TB) (rifampicin, rifabutin, isoniazid) was matched with a ratio of 1:3 based on a weighted algorithm, including body mass index (BMI) at sample date, age, gender, ethnicity, CD4 cell counts, HIV transmission group (men who have sex with men [MSM], heterosexual, other), and HIV RNA at the sample date. The summed weights concerning these six characteristics were based on a previous analysis in the same population (8). To exclude a diagnosis of active TB for the control population, we required a follow-up of at least 5 years after the respective sample date. Furthermore, we required that either both the case and the selected control have another opportunistic infection within 3 months of the sample date, or both have none. For each case sample, up to 3 control samples were selected. In order to balance potential MTB infection within the control population, around half of the controls were chosen having a positive IGRA or TST result (*control MTB+*), with the other half having negative IGRA or TST results (*control MTB*−).

### Sample preparation

The samples were ordered from the decentralized SHCS biobank and centrally processed at the University Hospital Zurich within 1 week by the same person using the PreOmics iST Kit in the 96-well format (11). The processing involved the addition of 4 µL of plasma and 100 µL of lysis buffer, followed by loading 50 µL onto the cartridge. On each batch, standardization samples were added as quality control to potentially detect plate differences consisting of a mixture of 2–5 µL of each included sample. Case and control samples were randomly assigned across batches (Fig. S1).

### Proteomics measurement

Quantitative data were acquired in dia-PASEF mode (data independent acquisition-parallel accumulation serial fragmentation) on a timsTOF Pro mass spectrometer equipped with the nanoElute Chromatography System (Bruker) (11, 12). This method uses trapped ion mobility separations to increase sensitivity and resolving power combined with data-independent acquisition to ensure robustness and data completeness. All analyses were performed using ~200 ng purified peptides. More details on the workflow can be found in the supplemental methods. To monitor the variation between sample batches and workflow steps, we employed two types of quality control (QC) samples: the first QC set consisted of a pooled sample (full process QC) included twice on every plate. The second QC set comprised a random mixture of 100 samples spiked with iRT peptides analyzed 78 times throughout the complete data acquisition process to assess instrument performance (measurement QC). These raw mass spectrometry (MS) data were monitored using QuiC software (Biognosys, Version 4) to ensure reproducibility of the QC indices. In addition, to assess potential issues with sample handling in the preparation phase, we calculated 3 quality measures suggested by Geyer et al.: the ratio of platelet and erythrocyte contamination as well as sample coagulation (13).

### Proteomics data analysis

Spectronaut (version 18.1) was used for comparative analysis to transform diaPASEF raw data into precursor and fragment identifications by deconvolution-based analysis. The spectral library was generated using all raw data and 14 IM-GPF data using the default settings and used for library-based search analysis. All data were searched against the human proteome sequence database downloaded from UniProt on 7 January 2022, comprising 20,387 entries, using trypsin as the digestion enzyme. Cysteine carbamidomethylation was set as a fixed modification, and methionine oxidation and excision at the N terminus were selected as variable modifications. A maximum of two missed cleavages and up to five variable modifications were allowed. The FDR cutoff was set to 1% at PSM, peptide, and protein levels. The cutoffs for precursor *q*-value, precursor peptide, and protein *q*-value (experiment and run) were all set to 0.01 (14). Protein inference used the IDPicker algorithm using peak area for quantification. Imputation and normalization were performed using default global imputing and cross-run normalization methods, respectively (15). Log10 transformation was applied to the data matrix containing quantity information of 1,412 different proteins for each sample.

### Statistical analysis

All analyses were carried out using R (version 4.3.1). The main steps were the following: (i) training and validation set: we randomly split participants with active TB (cases) and their matched controls into 1/3 validation and 2/3 training cases, with the corresponding matched controls comprising the validation and training data set; (ii) selection of candidate proteins: within the training set, potential candidate proteins for the development of a prognostic TB score were selected using a linear mixed-effects model (R package *lme4*), including a time trend concerning time to TB diagnosis; all proteins with a *P*-value of less than 0.03, unadjusted for multiple testing at this pre-selection step, either for the comparison case versus control or the interaction with time were selected as potential candidate proteins; with this threshold, we selected roughly 5% of the full library as potential candidates; (iii) development of a prognostic score: a random forest algorithm (R package *caret*) with fivefold cross-validation (i.e., further division of the training set), Cohen’s kappa as accuracy metric, and weighing of the cases with a ratio of 1:3 (reflecting the distribution of cases and controls) was applied using the pre-selected protein candidates; here, only one sample per patient was used, namely, the sample closest to the active TB diagnosis for cases; and (iv) validation of the prognostic score: the score was evaluated using the validation set to assess the final score performance; we used the area under the receiver operating characteristic (ROC) curve (AUC) as well as the area under the precision-recall curve (AUPRC) to assess precision in this imbalanced data set (ratio case versus control was around 1:3); in addition, the model performance stratified by MTB+ and MTB− controls (using *T* tests to evaluate differences in the score) was assessed, as well as the correlation of the timing of the sample and the score by using linear regression.

### Association of the score and clinical characteristics

The association of the score with the following demographic and clinical characteristics was assessed within the validation set: age, sex, ethnicity (white, black, other), body mass index, MTB status, diagnosis of additional opportunistic infections, and several laboratory parameters (HIV RNA, creatinine, leukocyte count, triglycerides, CD3, CD4, CD8 cell count, HDL, cholesterol, glucose).

### Comparison with existing scores

To understand the performance of the newly generated risk score compared to that of other published diagnostic and prognostic risk scores for active TB, we searched for published scores where all proteins were available in our 1,412-protein library. For each identified published score, we first re-trained a random forest model with restriction to proteins included in the published risk score using the same training set as before and evaluated the score on our validation set (i.e., a validation set not included for the generation of the model). We repeated this procedure for all identified published risk scores separately. We compared the performance of our developed score and the published scores using the AUC-ROC, as this was the metric usually assessed in other publications.

## RESULTS

### Patient characteristics

We identified 91 participants with a diagnosis of active TB, with a total of 178 available samples (68 in the range of 6–12 months, 62 in the range of 1–2 years, and 48 in the range of 2–4 years before active TB diagnosis). The control population comprised 293 SHCS participants with a total of 405 samples (Fig. S2). Most of the cases were male (58.2%) with a median age at diagnosis of 38 (interquartile range [IQR] = 31–43.5). Around half (46.1%) had a suppressed HIV RNA viral load (<400 copies/mL) at the sample date closest to TB diagnosis (Table 1).

**TABLE 1:** Basic characteristics of the included participants stratified by cases and controls

Parameter	Cases	Controls
Total no. of patients	91	293
Gender		
Male	53 (58.2%)	169 (57.7%)
Female	38 (41.8%)	124 (42.3%)
Ethnicity		
White	45 (49.5%)	183 (62.5%)
Black	34 (37.4%)	86 (29.4%)
Other	12 (13.2%)	24 (8.2%)
Risk group		
MSM	19 (20.9%)	73 (24.9%)
HET	49 (53.8%)	147 (50.2%)
IDU	20 (22%)	59 (20.1%)
Other	3 (3.3%)	14 (4.8%)
Age at sample date: median [IQR]	38 (31–43.5)	41 (36–44)
CD4 at sample date		
>500	22 (24.2%)	128 (43.7%)
350–500	25 (27.5%)	69 (23.5%)
200–350	19 (22%)	54 (19.1%)
<200	25 (27.5%)	42 (14.3%)
RNA at sample date		
Suppressed (<400 copies/mL)	42 (46.2%)	179 (61.1%)
>400 copies/mL	49 (53.8%)	114 (38.9%)
BMI		
<18.5	9 (9.9%)	21 (7.2%)
18.5–25	59 (64.8%)	187 (63.8%)
>25	21 (23.1%)	85 (29%)
Total no. of samples	178	405
Time period		
6–12 months	68 (38.2%)	165 (40.7%)
1–2 years	62 (34.8%)	155 (38.3%)
>2 years	48 (27%)	85 (21%)
Calendar year of samples: median [IQR]	2005 (2002–2009)	2007 (2004–2011)

### Plasma proteomics data

**Fig 2:**
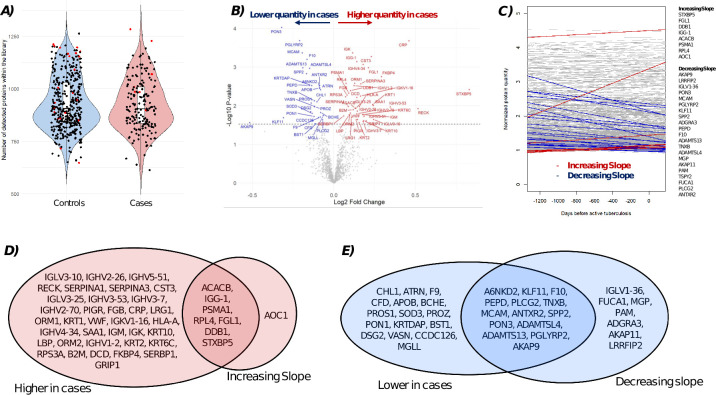
Information on proteins in the library. (**A**) Number of proteins detected per sample. The red points indicate the samples, which were outliers with respect to other quality metrics (see Fig. S3); (**B**) significant (*P* < 0.03) association between cases and controls determined via linear mixed-effects model; (**C**) time trend of all proteins with respect to time to diagnosis: each line shows the time trend of one protein; and (**D**) proteins showing higher or increased quantities, plus overlap; and (**E**) proteins showing lower or decreasing quantities, plus overlap.

The generated spectral library contained 19,041 peptide precursors for 1,412 proteins, from which 613 to 1,283 proteins per sample (median = 906, IQR = 838–1,021) were quantified across the full sample set (Fig. 2A). After reviewing all quality check procedures, all samples could be kept within the data set (Fig. S3 and S4).

### Proteomic signature of TB progression reveals enrichment in inflammatory and humoral immune pathways

**Fig 3:**
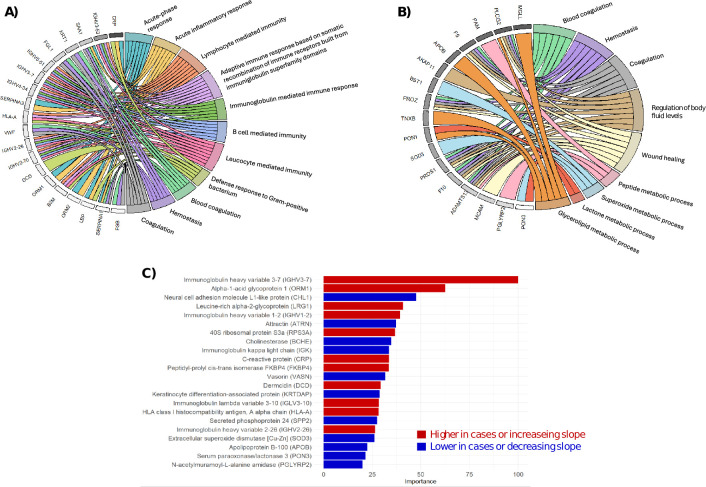
Importance of candidate proteins: (**A**) biological processes involved in proteins that are higher in cases or have an increasing slope toward diagnosis of active TB; (**B**) biological processes involved in proteins that are lower in cases or have a decreasing slope toward diagnosis of active TB; and (**C**) variable importance within the random forest algorithm.

We obtained 446 samples in the training set, for which 1,412 different proteins were measured. We chose the unadjusted *P*-value cut-off of 0.03 for a difference between cases and controls or a time trend, respectively, leading to 81 (5.7%) candidate proteins. Of these, 73 were below this chosen cut-off when comparing cases and controls, with 43 showing an upregulation and 30 showing a downregulation (Fig. 2B; Table S1). Furthermore, 29 proteins were below the chosen threshold concerning the time trend, with 21 showing an increase toward diagnosis and eight showing a decrease (Fig. 2C; Table S1). Enriched pathways included acute-phase response, blood coagulation, and wound healing (Fig. 3A through C).

Figure 3 illustrates the functional interpretation of these candidate proteins. Panels (**A**) and (**B**) show chord diagrams mapping proteins with higher abundance in cases or increasing toward TB diagnosis (**A**) and those lower in cases or decreasing (**B**) to Gene Ontology biological processes. Upregulated proteins were strongly linked to acute inflammatory responses, B cell-mediated immunity, immunoglobulin production, and defense responses to pathogens. In contrast, downregulated proteins were associated with metabolic processes, wound healing, and hemostasis.

Panel (c) ranks the top 20 proteins by importance in the random forest classifier. Notably, several immunoglobulin heavy and light chain variants featured prominently among the most predictive proteins, reinforcing a central role of humoral immunity in TB progression.

### Prognostic score for active TB

**Fig 4:**
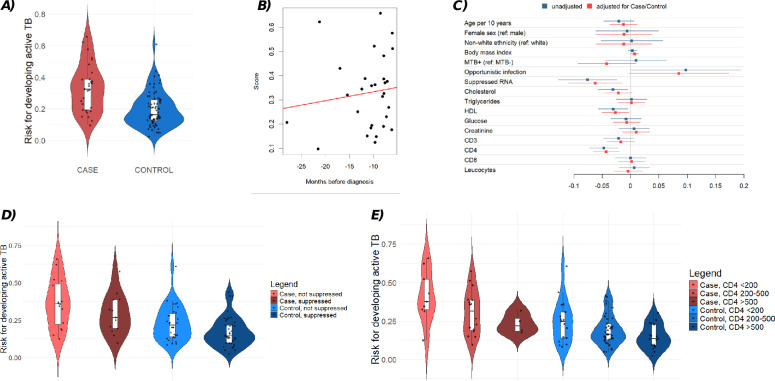
Performance of the risk score within the test set: (**A)** case versus controls, (**B**) risk score within cases over time, (**C**) clinical and demographic characteristics associated with the risk score, first unadjusted for case/control status and second adjusted for case/control status, (**D**) stratification by HIV viral load suppression, and (**E**) stratification by CD4 cell count.

A random forest algorithm was developed using 81 candidate proteins and 284 training set samples, selecting one sample per individual (the sample closest to the diagnosis date for cases). The model was validated on a separate data set including 100 samples. The median score for cases progressing to active TB (i.e., the median probability for a case sample progressing to active TB as predicted by the random forest classifier) was 0.33 (IQR: 0.19–0.39) compared to 0.16 (IQR: 0.13–0.26) for controls (*P* < 0.001), indicating a more than twofold higher predicted risk for TB progression among cases (Fig. 4A). This approximate twofold difference was robust when evaluating samples within 6–12 months (0.34, IQR: 0.19–0.39) and those more than 1 year (0.30, IQR: 0.20–0.41) before diagnosis of active TB separately, although with small sample sizes per category (Fig. S5). Similarly, there was only a slight increase but no significant relationship between the time now as a continuous variable from sample collection to TB diagnosis and the score among 29 test set case samples (*P* = 0.52, Fig. 4B). However, including all available samples (i.e., training and validation set [*N* = 583]) did reveal a trend of an increasing score within the same individuals toward TB diagnosis (Fig. S6). Notably, the score did not differ significantly between MTB+ (median: 0.15, IQR: 0.10–0.24) and MTB− (median: 0.17, IQR: 0.13–0.27) controls (*P* = 0.28).

In terms of the predictive power of the risk score, we obtained an AUC-ROC of 0.77 and an AUPRC of 0.60 (as compared to an expected AUPRC of 0.29, as 29 cases are among the 100 control samples). The optimal threshold on the ROC curve was 0.311, reaching a specificity of 87.3% and a sensitivity of 58.6%.

### External validation of previously identified candidate proteins

**Fig 5:**
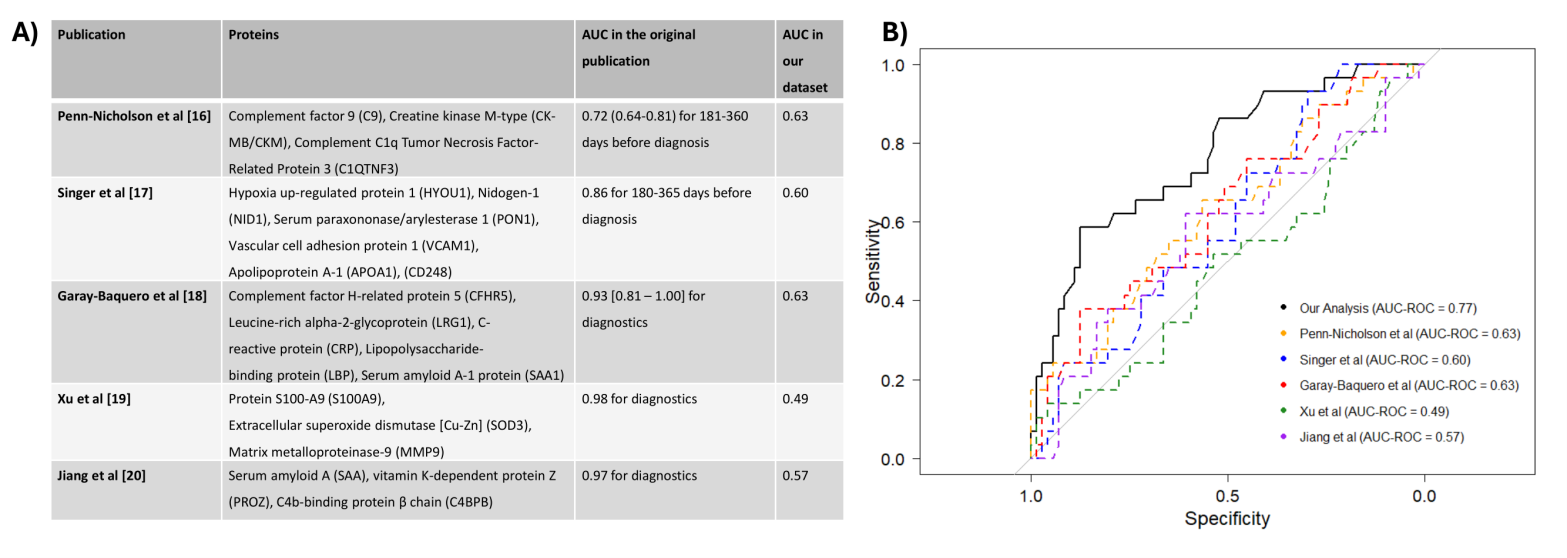
Performance of other candidate protein sets within our sample set. (**A**) The five investigated publications are listed in bold overlapping proteins within our 81-candidate set. The AUC-ROC reported in the original publication as well as the AUC-ROC of predicting active TB within our validation set are included in the third and fourth columns, respectively; and (**B**) visualization of the receiver operating characteristics curves (ROC).

We identified several previously published protein-based predictive scores with included proteins present in our library (Fig. 5). First, we assessed the three-protein signature developed by Penn-Nicholson et al. based on 46 cases and 106 control samples from an HIV-uninfected adolescent population in South Africa (16). None of the three proteins were among our 81 selected candidate proteins, yet the predictive performance yielded an AUC of 0.63 in our data set compared to 0.72 (0.64–0.81) for 181–360 days before diagnosis in the original publication. Second, we evaluated the candidate proteins from the six-protein prognostic score in the South Africa cohort (*N* = 30) developed by Singer et al. in a population of PWH. While in the original population, the prognostic performance was reported as an AUC of 0.86 for 180–365 days before diagnosis, we achieved an AUC of 0.60 in our validation set (17). Third, we examined the five-protein panel developed by Garay-Baquero et al., which was based on 11 patients without HIV and 10 healthy controls from South Africa and Peru (18). Four of the five proteins were also identified in our candidate set, yielding an AUC of 0.63 in our validation set. Next, we tested the three-protein panel by Xu et al., which was developed from 40 cases and 40 controls in China (19). Only one of these proteins overlapped with our candidate set, resulting in a limited predictive performance of AUC = 0.49 in our data set. Finally, we evaluated the three-protein biomarker identified by Jiang et al. based on 136 cases and 66 controls in China (20). Two of the three proteins overlapped with our candidate set, achieving an AUC of 0.57 in our validation set. The last three reported scores were only assessed for diagnostic performance in the original publications; thus, the direct comparison with our obtained AUCs for prognostic purposes should not be overinterpreted.

### Correlation with patient characteristics

Among the clinical and demographic variables tested in the validation cohort, CD4 count (*P* = 0.0004), suppressed HIV RNA (*P* = 0.02), cholesterol (*P* = 0.02), and HDL (*P* = 0.02) were each significantly associated with the proteomic risk score (Fig. 4C). These associations remained largely significant after adjusting for case/control status, particularly for CD4 count (*P* = 0.0003), suppressed HIV RNA (*P* = 0.01), and HDL (*P* = 0.03). Importantly, the risk score showed consistent shifts across categories of CD4 count and viral suppression status (Fig. 4D and E), indicating that the score reflects established clinical indicators. The most revealing pattern emerged when comparing groups with mismatched clinical and virologic profiles. Cases with suppressed HIV RNA and viremic controls had similar risk scores. However, the score remained distinctly higher in cases than in controls when both groups had suppressed HIV RNA. This distinction highlights the added value of plasma proteomics: it reveals biological differences that are not captured by standard clinical measurements alone.

The highly granular clinical phenotype data available within the SHCS allows us in addition to retrospectively investigating the characteristics of individual patients, introducing substantial additional value by helping us to understand why the score misclassified certain controls. For example, one control patient had a risk score of greater than 0.6 for developing active TB (Fig. 4A). This patient had a CD4 cell count of only 59 cells/µL and a concurrent herpes zoster infection, both indicative of significant immune suppression. Additionally, three control patients with risk scores between 0.4 and 0.5 had CD4 counts of 117, 364, and 280 cells/µL, respectively. For reference, the mean CD4 count among control samples was 380 cells/µL, highlighting that lower CD4 counts are associated with higher TB risk scores.

Furthermore, two patients had infections with *Mycobacterium avium*: one control patient with a risk score of 0.36 and one case patient co-infected with both *Mycobacterium tuberculosis* and *M. avium* who had a risk score of 0.38. These scores were significantly higher than the median risk score in the validation data set, which was 0.21, suggesting that immune suppression and the presence of any opportunistic infection may contribute to elevated risk scores.

## DISCUSSION

Technical advances of high-throughput proteomics and novel computational tools offer promising solutions for the development of new biomarkers, such as urgently needed alternatives to T-cell-based TB diagnostics (16, 21, 22). In order to apply these new methods, however, well-characterized pre-disease onset samples and long follow-up durations are necessary. In this study, we leveraged longitudinally and routinely collected samples and data within the SHCS to develop a proteome TB risk score in PWH. With robust case-control matching, including viremic individuals, we were able to assess the predictive power of this score specific to TB, even including temporal variation with respect to the time point of active TB.

Among the most striking findings of our analysis was the consistent association of B cell responses and immunoglobulin production with progression to active tuberculosis. While historically the role of antibodies in TB has been considered limited, emerging evidence challenges this paradigm. A landmark study demonstrated that individuals with latent TB produce antibodies with distinct Fc functional profiles and glycosylation patterns compared to those with active TB, suggesting that protective antibody responses may shape disease outcomes (23). More recently, TB-specific antibodies were shown to exhibit unique effector functions and subclass distributions depending on the clinical disease state, further supporting the concept that qualitative features of humoral immunity influence TB pathogenesis. Additional studies have emphasized the immunomodulatory role of B cells and antibodies in TB through mechanisms, including opsonization, antigen presentation, and inflammatory regulation (24–26). In HIV-TB co-infection, where T cell immunity is often compromised, antibody-mediated mechanisms may become even more relevant, though they remain underexplored. HIV-associated B cell dysfunction is known to alter antibody quality and repertoire, which could influence TB susceptibility and progression (27). Our findings strongly suggest that, even in people with HIV, humoral immune perturbations—particularly involving B cell activation and immunoglobulin production—are key features of TB progression (28). This supports a growing recognition of the role of antibodies in TB and HIV-TB co-infection (29).

Our plasma proteome score demonstrated a performance comparable to or better than other published biomarkers in predicting active TB. While some studies have reported very high performance for distinguishing active TB from healthy controls, these results must be interpreted cautiously. Factors, such as study setting, sample availability, and the methods used to calculate the scores, play a critical role in performance estimates. To evaluate how our newly developed score compares to existing proteome-based biomarkers, we identified five studies with published scores that included proteins present in our library. For example, four out of five proteins of the five-protein risk score developed by Garay-Baquero et al. were overlapping with our identified proteins, but nevertheless showed limited predictive power within our data set (18). This indicates potential limitations concerning reproducibility of such scores; however, we observed a significant improvement in performance when adding additional proteins. Similar findings were observed for the other protein biomarkers we investigated. This analysis of using our data set as an external validation of published risk scores, thus, suggests that the predictive power of risk scores including only a small set of proteins is most likely limited. Although some single proteins showed some predictive power, including also proteins previously identified in other publications, the combination of all 81 proteins outperformed this predictive performance, suggesting that expanding the protein panel enhances predictive accuracy and robustness.

Interestingly, our proteome score shows no strong evidence of time dependency over the observation period. This is in contrast to published transcriptome-based scores, which showed increasing trends as the sample date approaches the diagnosis of clinically overt disease, potentially driven by type 1 interferon-driven (30). This indicates that the proteome score is less likely to diagnose early disease and instead reflects the overall immune status. This is also seen in the regression analysis, which revealed an inverse correlation between our risk score and CD4 cell counts and the occurrence of opportunistic infections, suggesting that the score provides a proxy for overall immune status. TB often co-occurs with other opportunistic infections in PWH, and our analysis highlights the multifaceted nature of the score. Notably, two individuals with *M. avium* infection exhibited particularly high-risk scores, with one subsequently developing active TB, while the other did not.

Choosing the optimal threshold, our newly developed risk score reaches a sensitivity of 58.6% and a specificity of 87.3%. A recent publication within the same study population estimated a sensitivity of only 20% and a specificity of 95% of the current gold standard in terms of predicting progression to active TB at least 6 months before diagnosis (8). The high sensitivity of our newly developed risk score is particularly remarkable given that the case and control population was matched by demographic and clinical characteristics, in particular ethnicity, CD4 cell count, and HIV RNA suppression, factors which are independently associated with risk for developing active TB. In another recent publication within our group, a machine learning algorithm for predicting active TB was developed using clinical and demographic parameters only, reaching a sensitivity of 70.1% and a specificity of 81.0% (AUC-ROC: 0.83) (31). Given the promising predictive power of both—only clinical and demographic values or only information given by the proteome (with other factors matched)—suggests that combining clinical and demographic information with proteomics might even yield better results.

This study has several strengths and limitations. A major strength is the clear phenotype definition for active TB and the longitudinal and retrospective design enabling the selection of control participants who did not progress to active TB over a follow-up period of more than 5 years. This, to our knowledge, represents the most stringent criterion for a control population used in the development of prognostic (or diagnostic) TB risk scores. A further strength of our study is the use of TB-negative and MTB-positive controls, where we observed surprisingly no significant difference between the two groups. This control MTB+ population in our study is distinctive, as MTB infection preventive measures were not applied as indicated by established guidelines, making them an understudied group. This provides a unique opportunity to differentiate true progression to active TB from MTB infection, offering valuable insights for future studies. We initially aimed to create a TB-specific proteomic risk score by carefully matching cases and controls on key clinical and demographic variables. While this matching strategy helped reduce confounding, it was not perfect—some factors strongly associated with active TB, such as low CD4 count or high HIV viral load remained unevenly distributed. Despite this, the analysis revealed that the risk score still correlated with CD4 count and HIV viral load independently of the TB case/control status. Specifically, lower CD4 counts and viremic HIV infection were each associated with higher risk scores, suggesting that the proteomic signal reflects the host immune status beyond the presence of active TB alone. One limitation of the study is inherent to the design, which required plasma samples to be collected at least 6 months before the diagnosis of active TB. This criterion excluded individuals with late HIV presentation and simultaneous TB diagnosis, thereby reducing the study population. Specifically, only 11% of participants with active TB met the criterion of diagnosis at least 6 months after enrolling in the SHCS with available plasma samples.

In summary, our study underscores the potential of proteomics as a robust approach for identifying biomarkers relevant to TB risk, particularly in the context of PWH. By leveraging a well-defined cohort with longitudinal sampling and stringent control criteria, we were able to develop a proteome-based score that not only predicts TB progression but also reflects a broader immune status. While the proteome score appears not to diagnose early subclinical TB, its association with immune suppression and opportunistic infections highlights its relevance in populations with complex immune dynamics. Future studies with larger sample sizes and diverse immune-suppressed patients will be essential to validate these findings and further refine the utility of proteomic risk scores for both TB-specific and general immune health applications.

## Data Availability

The Swiss HIV Cohort Study (SHCS) established in 1988 collects clinical data and plasma samples from people living with HIV in Switzerland. All participants have provided written informed consent; however, strict confidentiality rules apply. Specifically, the SHCS is obligated to ensure that individuals cannot be re-identified from shared data, which prevents the release of detailed participant-level data (https://www.shcs.ch/for-researchers/open-data-statement-shcs/). To enable transparency and reproducibility while respecting these privacy obligations, we provide the protein quantities plus key sample meta data (case/control status, timing of the sample, MTB status, train/validation-status) in the Supplemental Data. With these data, the reproducibility of the protein candidate pre-selection and development of the risk score are ensured. For projects that require participant-level data variables, a proposal needs to be submitted to the Scientific Board of the SHCS: https://www.shcs.ch/for-researchers/who-can-submit/.

## SUPPLEMENTAL MATERIAL

Supplemental Material mbio.01585-25-s0001.docx Supplemental methods, figures, and tables.

Data File mbio.01585-25-s0002.csv Information on the 583 samples.

ASM does not own the copyrights to Supplemental Material that may be linked to, or accessed through, an article. The authors have granted ASM a non-exclusive, world-wide license to publish the Supplemental Material files. Please contact the corresponding author directly for reuse.
