# Supplementary material for: Inflammation and B cell activation define a plasma proteome signature predicting tuberculosis in people with HIV
Source: mBio. 2025 Aug 28;16(10):e01585-25. doi: 10.1128/mbio.01585-25 (PMC12505960; doi:10.1128/mbio.01585-25)
Supplement: Supplemental Material — Supplemental methods, figures, and tables. [file mbio.01585-25-s0001.docx]

**Supplementary Material**

**Inflammation and B Cell Activation define a Plasma Proteome Signature predicting Tuberculosis in People with HIV**

**Kusejko et al.**

***Supplemental Methods:***

***Proteomics measurement***

As explained in the main manuscript, quantitative data was acquired in dia-PASEF mode (data independent acquisition – parallel accumulation serial fragmentation) on a timsTOF Pro mass spectrometer equipped with nanoElute chromatography system (Bruker) (1). This method uses trapped ion mobility separations to increase sensitivity and resolving power combined with data independent acquisition to ensure robustness and data completeness. All analyses were performed using ~200 ng purified peptides.

In more detail, the workflow was as follows: Peptides were separated on an integrated packed emitter column (10 cm × 75 μm ID, Coann) packed with 1.5 μm Reprosil Saphir beads (Dr. Maisch). The separation was performed using a gradient method: 5% to 24% Solvent B (0.1% formic acid in acetonitrile) over 15 minutes, followed by 33% Solvent B in 4 minutes, and then 95% Solvent B in 2 minutes and maintained at 95% Solvent B for 1 minute. The flow rate was set at 300 nL/min, and the column temperature was maintained at 50°C. Mass spectrometry data were acquired in dia-PASEF method optimized with py_diAID cover a mass-to-charge (m/z) range from 250 to 1300, including two IM windows per 8 dia-PASEF scans with variable isolation window widths adjusted to the precursor densities. The IM range was set to 1.40 and 0.68 V cm−2. The accumulation and ramp times were specified as 100 ms for all experiments. As a result, each MS1 scan and each MS2/dia-PASEF scan last 95 ms plus additional transfer time. The collision energy was decreased as a function of the IM from 59 eV at 1/K0 = 1.6 V cm−2 to 20 eV at 1/K0 = 0.6 V cm−2. The IM and m/z dimensions were calibrated with three Agilent ESI Tuning Mix ions (m/z, 1/K0: 622.02, 0.991 V cm−2, 922.01, 1.198 V cm−2, and 1221.99V, 1.393 V cm−2). A gas phase fractionation acquisition scheme called ion mobility gas phase fractionation (IM-GPF) were used for rapid dia-PASEF library generation (2). Briefly, data from seven injections of a representative sample in short LC method (as described before) and seven injections in long method (3% to 15% Solvent B over 40 min, followed by an increase to 25% over 25 min, then to 33% over 6 min, and finally to 95% over another 6 minutes) and were collected across a m/z range of 400–1200 using 5 m/z windows, each with a 1 m/z overlap on either side. The ion mobility spanned a range of 0.57–1.47 V/cm², with two quadrupole positions sampled per ion mobility cycle. These windows were evenly distributed across seven acquisition methods, with each method encompassing 15 ion mobility cycles.

***Figure S1:*** *Batch arrangement of the 583 samples*


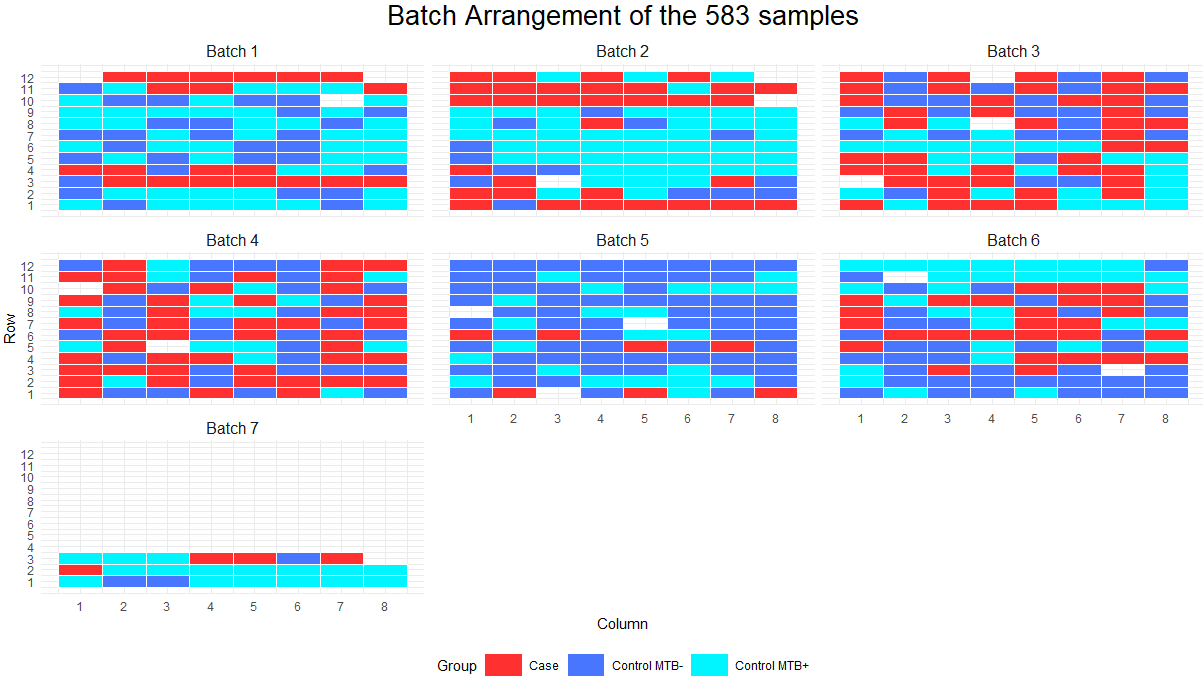


***Figure S2:*** *Timeline of all case and matched control samples.*


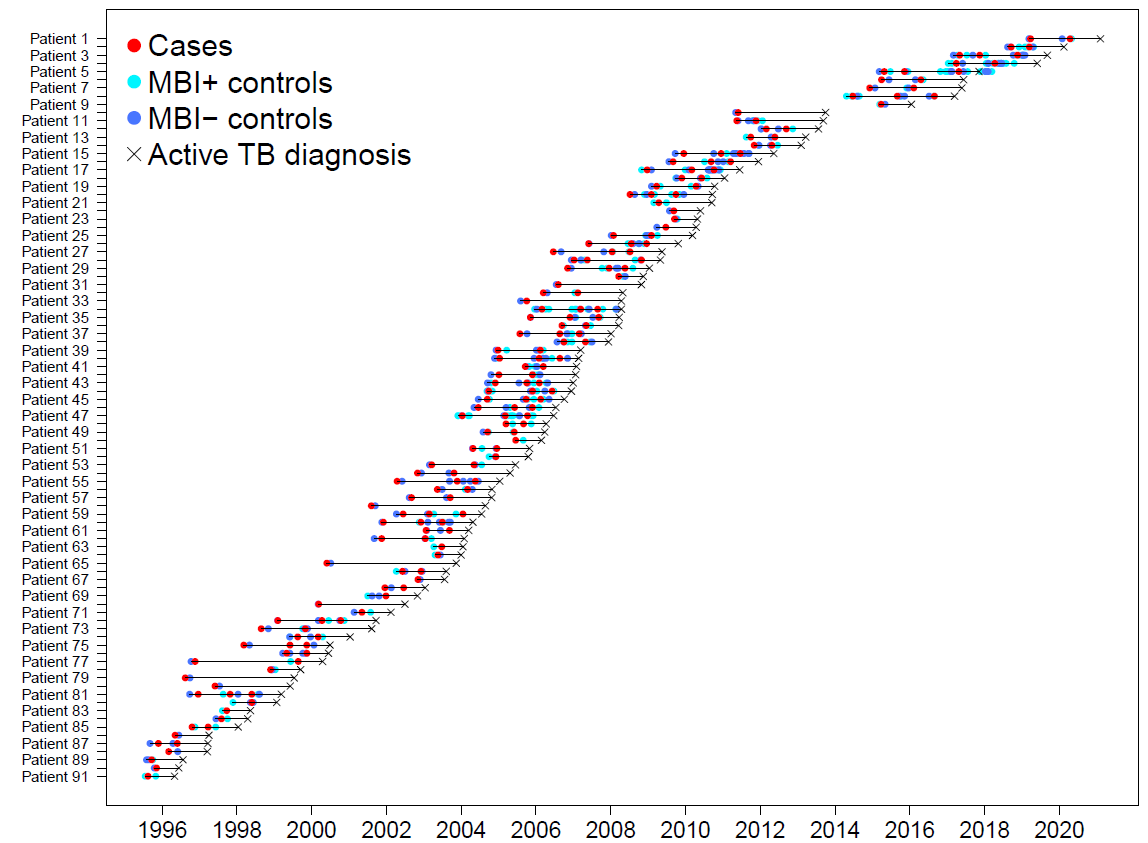


***Figure S3:*** *Quality control samples*

*A) Principal component analysis (PCA) of the three types of samples: Quality control (QC) for samples (in blue) and participant samples (in gray). It is seen that the biological variance across patient samples is substantially in excess of full process QC samples*


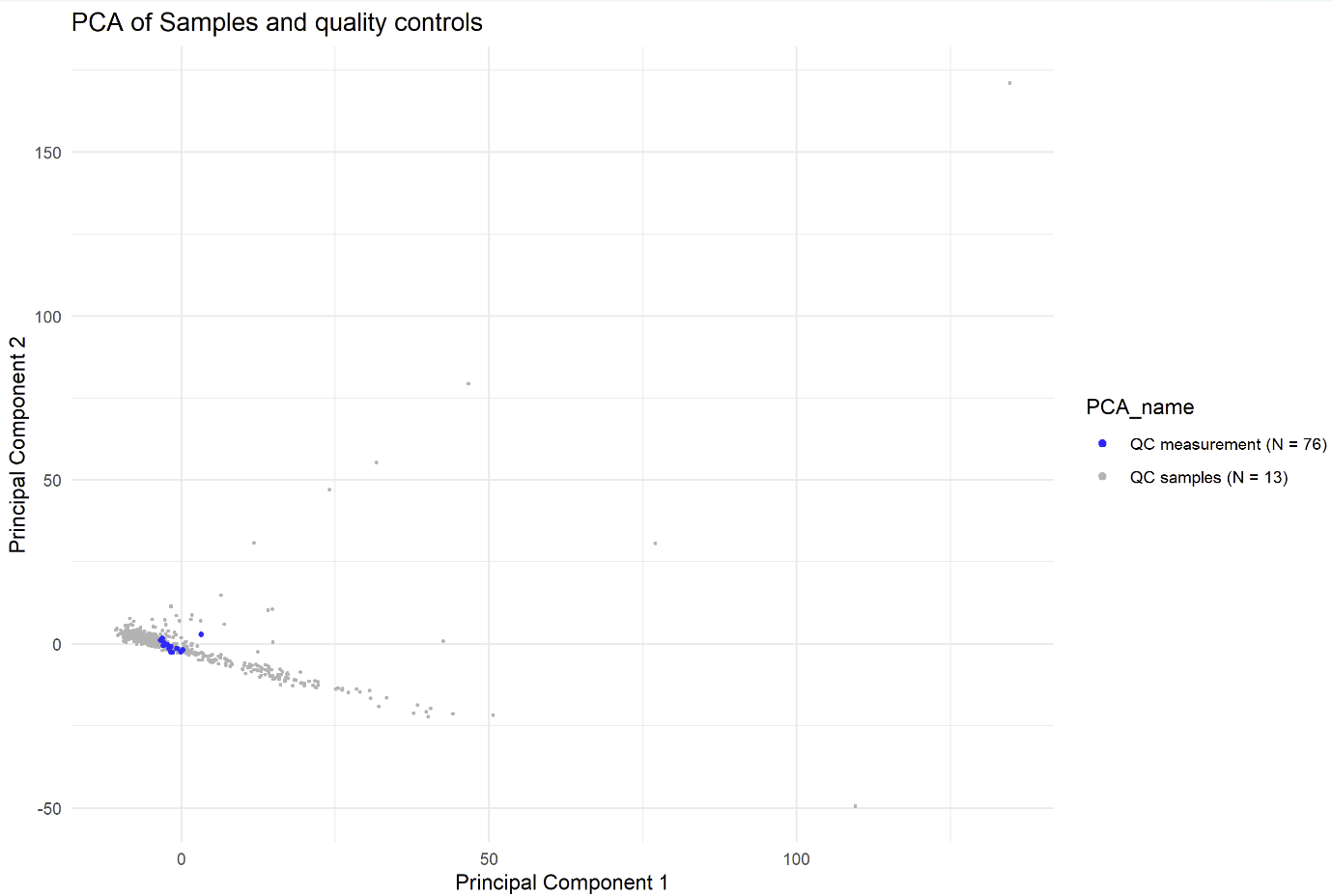


*B) Example of Measurement QC longitudinal evaluation: The measurement QC shows number of detected precursors and protein groups. This is representative of other QC measures evaluated (i.e. mass accuracy, retention time stability, ion mobility stability, peak area, etc – calculated by Quic software).* The yellow box indicates a maintenance period where LC column was replaced and no patient samples were acquired during this time.


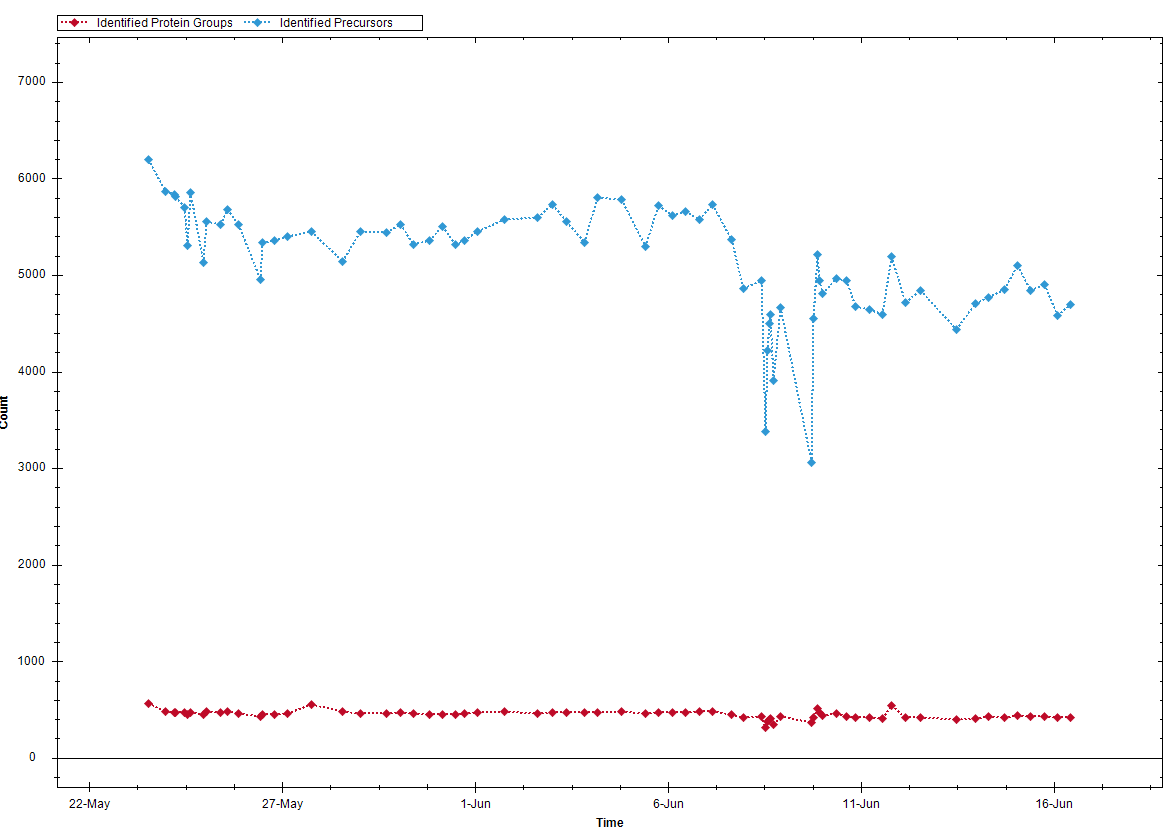


***Figure S4:*** *Quality assessment of the samples: Platelet contamination (A), Erythrocyte contamination (B), coagulation (C), and the fraction of detected proteins (D).*

*A: Assessment of platelet contamination, the horizontal line indicating a deviation three time the standard deviation.*


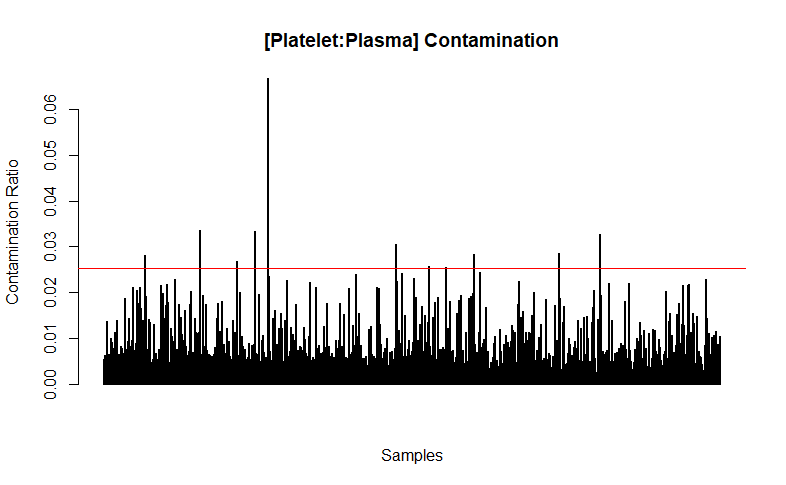


*B: Assessment of erythrocyte contamination, the horizontal line indicating a deviation three time the standard deviation*


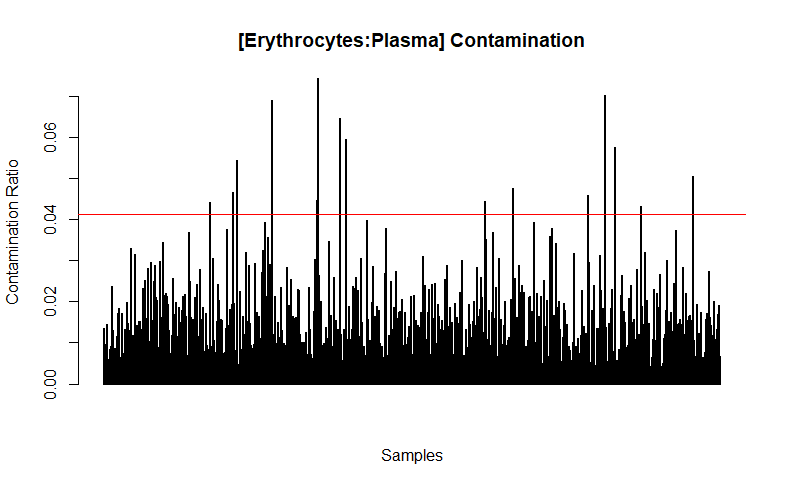


*C: Assessment of coagulation, the horizontal line indicating a deviation three time the standard deviation*


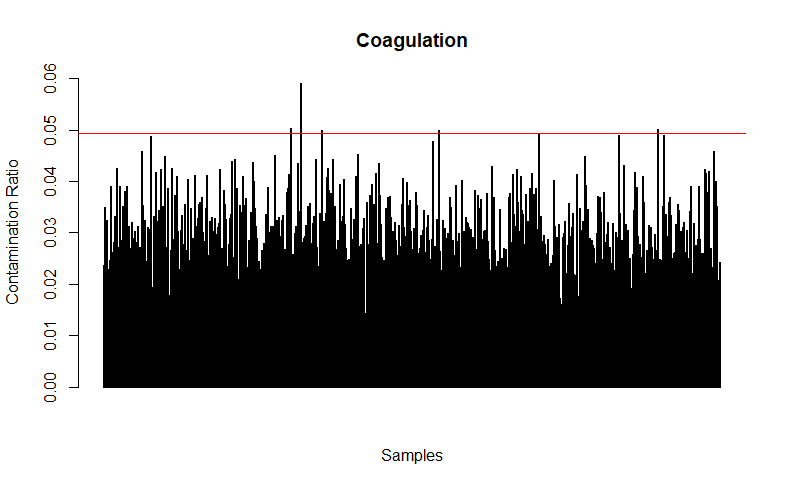


***Figure S5:*** *Risk score for developing active TB in the validation set, with case stratified by timing of the samples.*

***
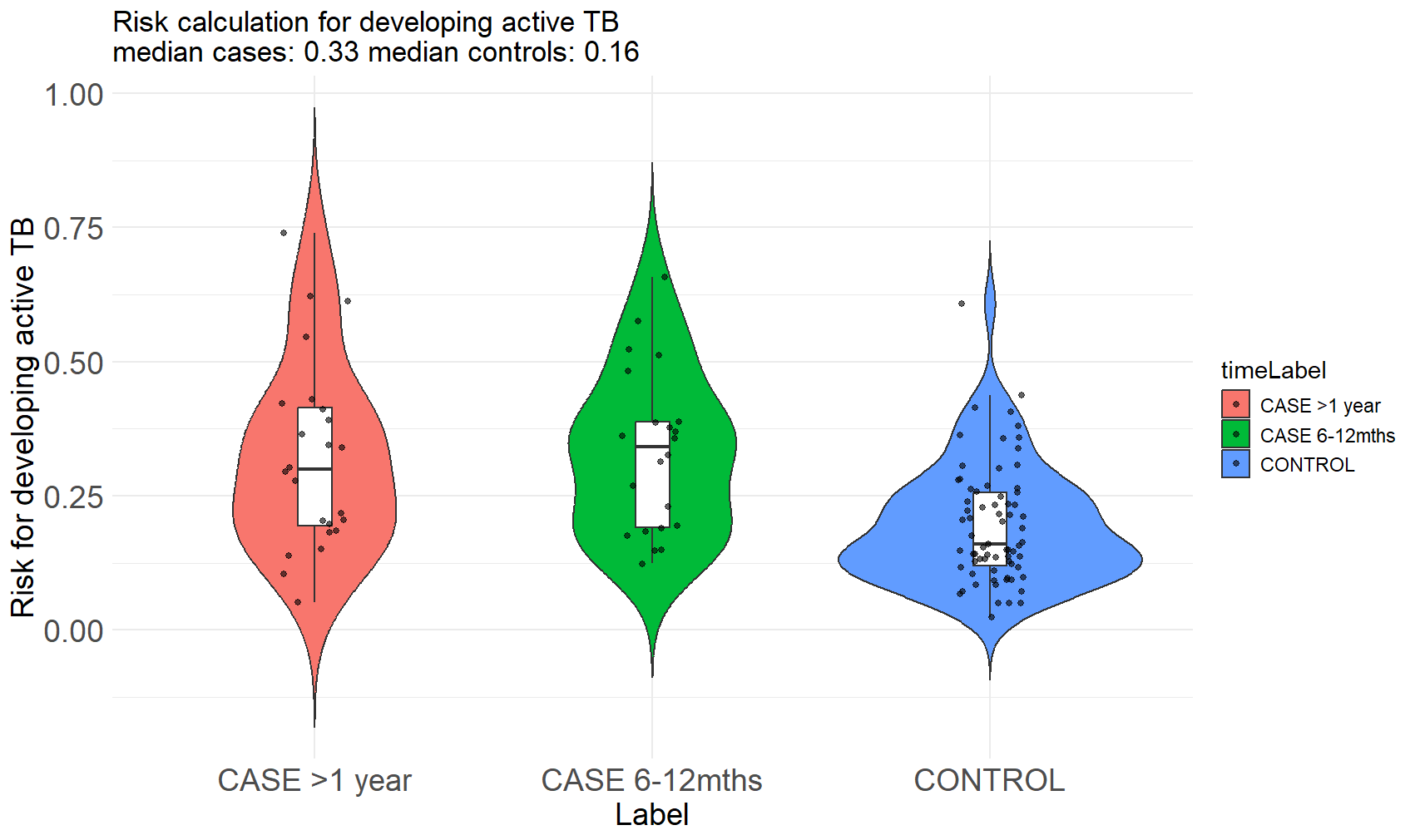
***

***Table S1:*** *Results of the mixed effects model for all 81 candidate proteins*

| **Label** | **Proteins** | **fold change** | **p case** | **slopes** | **p slope** |
| --- | --- | --- | --- | --- | --- |
| AKAP9 | A-kinase anchor protein 9 | -0.51773532 | 0.027344 | -0.00076330 | 0.005693 |
| PON3 | Serum paraoxonase/lactonase 3 | -0.32066542 | 9.47E-05 | -0.00045201 | 0.002537 |
| KLF11 | Krueppel-like factor 11 | -0.30980068 | 0.020434 | -0.00024991 | 0.008024 |
| IGLV1-36 | Immunoglobulin lambda variable 1-36 | -0.29045832 | 0.118488 | -0.00049197 | 0.011605 |
| LRRFIP2 | Leucine-rich repeat flightless-interacting protein 2 | -0.23409274 | 0.168953 | -0.00064824 | 0.027957 |
| PGLYRP2 | N-acetylmuramoyl-L-alanine amidase | -0.21039999 | 0.000208 | -0.00027201 | 0.002086 |
| MCAM | Cell surface glycoprotein MUC18 | -0.19438349 | 0.00043 | -0.00029825 | 0.001355 |
| ADAMTS13 | A disintegrin and metalloproteinase with thrombospondin motifs 13 | -0.19125637 | 0.001046 | -0.00018495 | 0.008156 |
| SPP2 | Secreted phosphoprotein 24 | -0.16883363 | 0.001485 | -0.00023681 | 0.016236 |
| F10 | Coagulation factor X | -0.16594364 | 0.000632 | -0.00021699 | 0.000552 |
| PEPD | Xaa-Pro dipeptidase | -0.15294785 | 0.002989 | -0.00021863 | 0.017247 |
| ADAMTSL4 | ADAMTS-like protein 4 | -0.14944011 | 0.001075 | -0.00013412 | 0.026392 |
| DSG2 | Desmoglein-2 | -0.14285538 | 0.014289 | -0.00018449 | 0.121093 |
| PROS1 | Vitamin K-dependent protein S | -0.14017141 | 0.008495 | -0.00016303 | 0.041206 |
| KRTDAP | Keratinocyte differentiation-associated protein | -0.1361513 | 0.003017 | -0.00019517 | 0.067775 |
| SOD3 | Extracellular superoxide dismutase [Cu-Zn] | -0.13410336 | 0.015823 | -0.00011140 | 0.187343 |
| ADGRA3 | Adhesion G protein-coupled receptor A3 | -0.12503284 | 0.106824 | -0.00023434 | 0.027854 |
| PON1 | Serum paraoxonase/arylesterase 1 | -0.12294364 | 0.019038 | -0.00015843 | 0.058636 |
| VASN | Vasorin | -0.12278196 | 0.010298 | -0.00008770 | 0.814033 |
| TNXB | Tenascin-X | -0.12025635 | 0.007463 | -0.00016570 | 0.008865 |
| PROZ | Vitamin K-dependent protein Z | -0.11779115 | 0.010584 | -0.00018336 | 0.134181 |
| CFD | Complement factor D | -0.11564131 | 0.029586 | -0.00015916 | 0.265029 |
| BST1 | ADP-ribosyl cyclase/cyclic ADP-ribose hydrolase 2 | -0.11116049 | 0.02967 | -0.00017425 | 0.110897 |
| CHL1 | Neural cell adhesion molecule L1-like protein | -0.10465649 | 0.007968 | -0.00016093 | 0.096457 |
| AKAP11 | A-kinase anchor protein 11 | -0.10290239 | 0.095543 | -0.00012094 | 0.026465 |
| CCDC126 | Coiled-coil domain-containing protein 126 | -0.09457265 | 0.020254 | -0.00012586 | 0.04359 |
| APOB | Apolipoprotein B-100 | -0.08955376 | 0.003504 | -0.00009105 | 0.15827 |
| ANTXR2 | Anthrax toxin receptor 2 | -0.08851349 | 0.002968 | -0.00007571 | 0.028447 |
| TSPY2 | Testis-specific Y-encoded protein 2 | -0.08740682 | 0.003471 | -0.00009377 | 0.001384 |
| F9 | Coagulation factor IX | -0.08400373 | 0.019693 | -0.00009289 | 0.393155 |
| ATRN | Attractin | -0.08316338 | 0.003886 | -0.00010909 | 0.172019 |
| BCHE | Cholinesterase | -0.0812739 | 0.01496 | -0.00007906 | 0.124096 |
| MGP | Matrix Gla protein | -0.08099736 | 0.081464 | -0.00012811 | 0.024501 |
| PAM | Peptidyl-glycine alpha-amidating monooxygenase | -0.07783904 | 0.047866 | -0.00011241 | 0.010021 |
| FUCA1 | Tissue alpha-L-fucosidase | -0.07077679 | 0.05875 | -0.00009098 | 0.02878 |
| PLCG2 | 1-phosphatidylinositol 4,5-bisphosphate phosphodiesterase gamma-2 | -0.06964723 | 0.023221 | -0.00008525 | 0.014126 |
| MGLL | Monoglyceride lipase | -0.06557472 | 0.018107 | -0.00005841 | 0.04691 |
| AOC1 | Amiloride-sensitive amine oxidase [copper-containing] | 0.03222519 | 0.034831 | 0.00006084 | 0.025753 |
| SERBP1 | Plasminogen activator inhibitor 1 RNA-binding protein | 0.059326865 | 0.018377 | 0.00007596 | 0.064754 |
| RPS3A | 40S ribosomal protein S3a | 0.071479923 | 0.00587 | 0.00008096 | 0.116966 |
| RPL4 | 60S ribosomal protein L4 | 0.073466377 | 0.002621 | 0.00008017 | 0.008442 |
| FGB | Fibrinogen beta chain | 0.080423649 | 0.005306 | 0.00007048 | 0.074565 |
| SERPINA1 | Alpha-1-antitrypsin | 0.087482858 | 0.010537 | 0.00007095 | 0.088433 |
| PSMA1 | Proteasome subunit alpha type-1 | 0.091230926 | 0.004691 | 0.00009688 | 0.00901 |
| LBP | Lipopolysaccharide-binding protein | 0.092944692 | 0.017375 | 0.00009210 | 0.262416 |
| ORM2 | Alpha-1-acid glycoprotein 2 | 0.100224371 | 0.015413 | 0.00010481 | 0.03433 |
| DDB1 | DNA damage-binding protein 1 | 0.1012019 | 0.004089 | 0.00015039 | 0.008876 |
| LRG1 | Leucine-rich alpha-2-glycoprotein | 0.106695154 | 0.019904 | 0.00008968 | 0.109611 |
| NA | Immunoglobulin kappa light chain | 0.114822436 | 0.000431 | 0.00010502 | 0.347885 |
| B2M | Beta-2-microglobulin | 0.117706791 | 0.012609 | 0.00007945 | 0.15542 |
| IGHV1-2 | Immunoglobulin heavy variable 1-2 | 0.123377822 | 0.003003 | 0.00005621 | 0.593886 |
| ORM1 | Alpha-1-acid glycoprotein 1 | 0.127358599 | 0.002624 | 0.00009132 | 0.117469 |
| ACACB | Acetyl-CoA carboxylase 2 | 0.129538773 | 0.011524 | 0.00013720 | 0.006702 |
| IGLV3-25 | Immunoglobulin lambda variable 3-25 | 0.133705511 | 0.01166 | 0.00010002 | 0.933691 |
| PIGR | Polymeric immunoglobulin receptor | 0.136020738 | 0.023173 | -0.00000835 | 0.639419 |
| DCD | Dermcidin | 0.139861197 | 0.006093 | 0.00014111 | 0.083389 |
| NA | Immunoglobulin gamma-1 heavy chain | 0.140078058 | 0.000722 | 0.00015007 | 0.002403 |
| IGHV2-70 | Immunoglobulin heavy variable 2-70 | 0.140272178 | 0.016229 | 0.00017931 | 0.112847 |
| IGHV2-26 | Immunoglobulin heavy variable 2-26 | 0.14359204 | 0.013788 | 0.00007733 | 0.958649 |
| VWF | von Willebrand factor | 0.144205174 | 0.022603 | 0.00002459 | 0.084908 |
| KRT2 | Keratin, type II cytoskeletal 2 epidermal | 0.15441495 | 0.027691 | 0.00012599 | 0.498236 |
| HLA-A | HLA class I histocompatibility antigen, A alpha chain | 0.156131425 | 0.010545 | -0.00002139 | 0.876428 |
| IGKV1-16 | Immunoglobulin kappa variable 1-16 | 0.162750905 | 0.005631 | 0.00000108 | 0.180046 |
| SERPINA3 | Alpha-1-antichymotrypsin | 0.16511006 | 0.002729 | 0.00012205 | 0.154455 |
| NA | Immunoglobulin mu heavy chain | 0.175248677 | 0.017037 | 0.00006880 | 0.595557 |
| IGHV4-34 | Immunoglobulin heavy variable 4-34 | 0.179192321 | 0.000856 | 0.00012578 | 0.639463 |
| IGHV3-7 | Immunoglobulin heavy variable 3-7 | 0.179336894 | 0.025525 | -0.00001954 | 0.849672 |
| KRT10 | Keratin, type I cytoskeletal 10 | 0.182915375 | 0.024559 | 0.00012436 | 0.431767 |
| KRT6C | Keratin, type II cytoskeletal 6C | 0.183481155 | 0.016887 | 0.00011128 | 0.468289 |
| IGHV5-51 | Immunoglobulin heavy variable 5-51 | 0.18397175 | 0.018888 | 0.00013411 | 0.303757 |
| GRIP1 | Glutamate receptor-interacting protein 1 | 0.196883634 | 0.024716 | 0.00025994 | 0.038558 |
| FGL1 | Fibrinogen-like protein 1 | 0.210713168 | 0.001047 | 0.00017598 | 0.027048 |
| CST3 | Cystatin-C | 0.229216671 | 0.000538 | 0.00010593 | 0.03009 |
| KRT1 | Keratin, type II cytoskeletal 1 | 0.238182058 | 0.009303 | 0.00014760 | 0.240871 |
| SAA1 | Serum amyloid A-1 protein | 0.24080345 | 0.009865 | 0.00019146 | 0.291575 |
| IGLV3-10 | Immunoglobulin lambda variable 3-10 | 0.24359479 | 0.022663 | 0.00005844 | 0.922526 |
| FKBP4 | Peptidyl-prolyl cis-trans isomerase FKBP4 | 0.291330991 | 0.001778 | 0.00013436 | 0.085871 |
| IGHV3-53 | Immunoglobulin heavy variable 3-53 | 0.345276531 | 0.010722 | 0.00023473 | 0.086904 |
| CRP | C-reactive protein | 0.45934438 | 0.000209 | 0.00039493 | 0.039863 |
| RECK | Reversion-inducing cysteine-rich protein with Kazal motifs | 0.515774976 | 0.011864 | 0.00033335 | 0.041313 |
| STXBP5 | Syntaxin-binding protein 5 | 0.830316872 | 0.003848 | 0.00105695 | 0.008174 |

***Figure S6:*** *Time trend of the score within the individuals; red lines indicate cases, black lines indicate controls.*

***
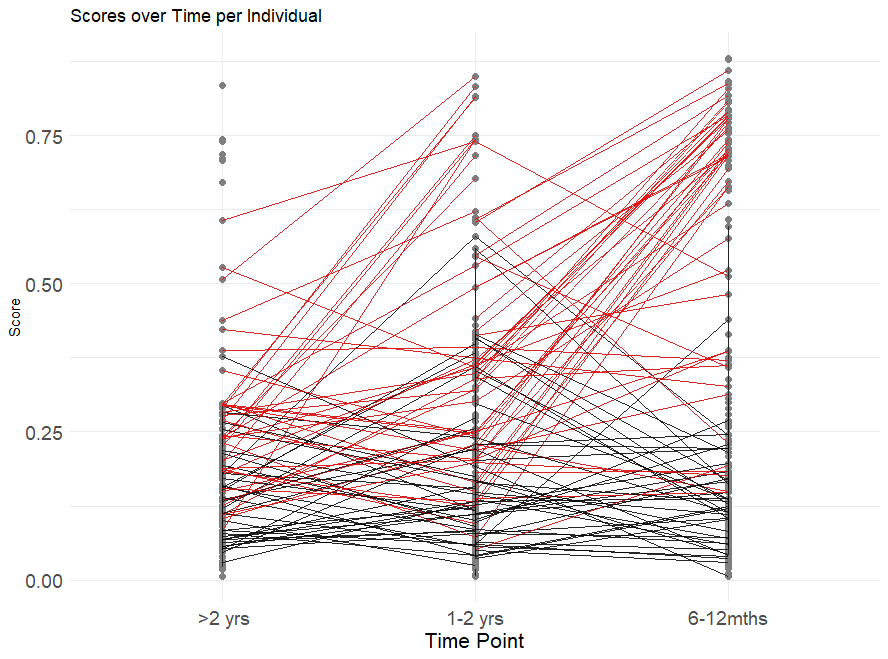
***

***References***

1. Kulak NA, Pichler G, Paron I, Nagaraj N, Mann M. Minimal, encapsulated proteomic-sample processing applied to copy-number estimation in eukaryotic cells. Nat Methods. 2014 Mar;11(3):319–24.

2. Penny J, Arefian M, Schroeder GN, Bengoechea JA, Collins BC. A gas phase fractionation acquisition scheme integrating ion mobility for rapid diaPASEF library generation. PROTEOMICS. 2023;23(7–8):2200038.
